# Potassium nickel(II) gallium phosphate hydrate, K[NiGa_2_(PO_4_)_3_(H_2_O)_2_]

**DOI:** 10.1107/S1600536809015438

**Published:** 2009-04-30

**Authors:** Ann M. Chippindale, Arun V. Sharma, Simon J. Hibble

**Affiliations:** a Department of Chemistry, University of Reading, Whiteknights, Reading, Berks RG6 6AD, England

## Abstract

The title compound, potassium nickel(II) digallium tris­(phosphate) dihydrate, K[NiGa_2_(PO_4_)_3_(H_2_O)_2_], was synthesized hydro­thermally. The structure is constructed from distorted *trans*-NiO_4_(H_2_O)_2_ octa­hedra linked through vertices and edges to GaO_5_ trigonal bipyramids and PO_4_ tetra­hedra, forming a three-dimensional framework of formula [NiGa_2_(PO_4_)_3_(H_2_O)_2_]^−^. The K, Ni and one P atom lie on special positions (Wyckoff position 4*e*, site symmetry 2). There are two sets of channels within the framework, one running parallel to the [10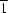] direction and the other parallel to [001]. These inter­sect, forming a three-dimensional pore network in which the water mol­ecules coordinated to the Ni atoms and the K^+^ ions required to charge balance the framework reside. The K^+^ ions lie in a highly distorted environment surrounded by ten O atoms, six of which are closer than 3.1Å. The coordinated water mol­ecules are within hydrogen-bonding distance to O atoms of bridging Ga—O—P groups.

## Related literature

For reviews of open-framework phosphate materials, see: Cheetham *et al.* (1999 ▶); Harrison (2002 ▶); Maspoch *et al.* (2007 ▶). For background to heterometal-substituted gallophosphates, *M*GaPOs, see: Baerlocher *et al.* (2001 ▶); Lin & Wang (2005 ▶). For related octa­hedral-trigonal bipyramidal silicate structures, see: Rocha & Lin (2005 ▶). For ammonium gallophosphates isostructural to the title compound, see: Chippindale *et al.* (1996 ▶, 1998 ▶); Bieniok *et al.* (2008 ▶). For the aluminium analogue, K[NiAl_2_(PO_4_)_3_(H_2_O)_2_], see: Meyer & Haushalter (1994 ▶). The same structure type occurs in Cs[Fe_3_(PO_4_)_3_(H_2_O)_2_] (Lii & Huang, 1995 ▶) and NH_4_[CoAl_2_(PO_4_)_3_(H_2_O)_2_] (Panz *et al.*, 1998 ▶) and is related to that of (NH_4_)_3_Ga_2_(PO_4_)_3_ (Lesage *et al.*, 2004 ▶). For bond-valence sums, see: Brese & O’Keeffe (1991 ▶); For the weighting scheme, see: Prince (1982 ▶); Watkin (1994 ▶).

## Experimental

### 

#### Crystal data

K[NiGa_2_(PO_4_)_3_(H_2_O)_2_]
*M* *_r_* = 558.17
Monoclinic, 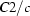

*a* = 13.2095 (13) Å
*b* = 10.1733 (9) Å
*c* = 8.8130 (9) Å
β = 107.680 (10)°
*V* = 1128.4 (2) Å^3^
*Z* = 4
Mo *K*α radiation
μ = 7.27 mm^−1^
*T* = 150 K
0.09 × 0.08 × 0.06 mm

#### Data collection

Oxford Diffraction Xcalibur diffractometer
Absorption correction: multi-scan (*CrysAlis RED*; Oxford Diffraction, 2006 ▶) *T*_min_ = 0.54, *T*_max_ = 0.65
3597 measured reflections
1913 independent reflections
1734 reflections with *I* > 2σ(*I*)
*R*_int_ = 0.018

#### Refinement

*R*[*F* ^2^ > 2σ(*F* ^2^)] = 0.022
*wR*(*F* ^2^) = 0.025
*S* = 1.04
1667 reflections
103 parameters
3 restraints
Only H-atom coordinates refined
Δρ_max_ = 0.84 e Å^−3^
Δρ_min_ = −0.91 e Å^−3^

### 

Data collection: *CrysAlis Pro* (Oxford Diffraction, 2006 ▶); cell refinement: *CrysAlis RED* (Oxford Diffraction, 2006 ▶); data reduction: *CrysAlis RED* (Oxford Diffraction, 2006 ▶); program(s) used to solve structure: *SIR92* (Altomare *et al.*, 1994 ▶); program(s) used to refine structure: *CRYSTALS* (Betteridge *et al.*, 2003 ▶); molecular graphics: *CAMERON* (Watkin *et al.*, 1996 ▶); software used to prepare material for publication: *CRYSTALS*.

## Supplementary Material

Crystal structure: contains datablocks global, I. DOI: 10.1107/S1600536809015438/fi2077sup1.cif

Structure factors: contains datablocks I. DOI: 10.1107/S1600536809015438/fi2077Isup2.hkl

Additional supplementary materials:  crystallographic information; 3D view; checkCIF report

## Figures and Tables

**Table 1:** Selected geometric parameters (Å, °)

Ga1—O1	1.8556 (11)
Ga1—O3	1.9994 (11)
Ga1—O5	1.8541 (10)
Ga1—O6	1.9455 (11)
Ga1—O7	1.8357 (11)
Ni1—O2	1.9951 (11)
Ni1—O2^i^	1.9951 (11)
Ni1—O3	2.1374 (11)
Ni1—O3^i^	2.1374 (11)
Ni1—O4	2.1030 (12)
Ni1—O4^i^	2.1030 (12)
P1—O1^ii^	1.5279 (11)
P1—O1^iii^	1.5279 (11)
P1—O3	1.5452 (11)
P1—O3^i^	1.5452 (11)
P2—O2	1.5135 (11)
P2—O5^iv^	1.5510 (11)
P2—O6^v^	1.5348 (12)
P2—O7	1.5434 (12)

Symmetry codes: (i) 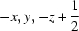; (ii) 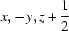; (iii) 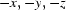; (iv) 
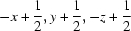
; (v) 
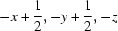
.

**Table 2:** Hydrogen-bond geometry (Å, °)

*D*—H⋯*A*	*D*—H	H⋯*A*	*D*⋯*A*	*D*—H⋯*A*
O4—H1⋯O5^ii^	0.85 (1)	1.896 (11)	2.741 (2)	172
O4—H2⋯O6^iv^	0.85 (1)	2.163 (17)	2.976 (2)	161

Symmetry codes: (ii) 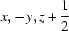; (iv) 
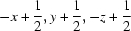
.

**Fig. 1.**
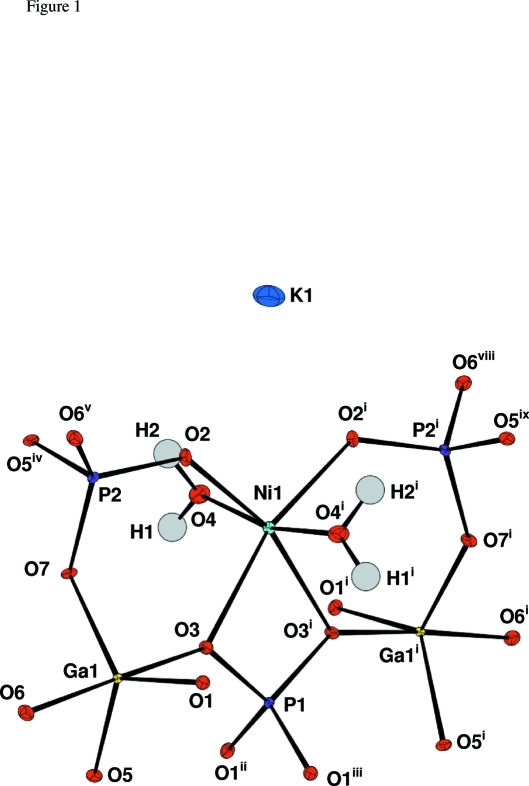
Local coordination of the title compound with displacement ellipsoids drawn at the 50% probability level. H atoms are shown as spheres of arbitary radius. [Symmetry codes: (i) -x, y, -z + 1/2; (ii) x, -y, z + 1/2; (iii) -x, -y, -z; (iv) -x + 1/2, y + 1/2, -z + 1/2; (v) -x + 1/2, -y + 1/2, -z; (viii) x - 1/2, 1/2 - y, 1/2 + z; (ix) -x + 1/2, y + 1/2, z].

**Fig. 2.**
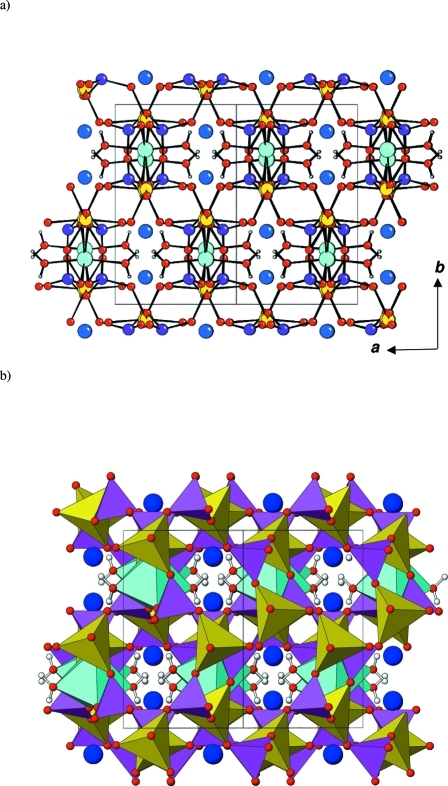
View of the main channels running parallel to the [1 0 1] direction shown as (a) ball and stick and (b) polyhedral representation. The cross pore distance O1···O5x is 7.251 (1) Å. The colour of each polyhedron corresponds to the colour of the central atom as defined in Fig. 1. [Symmetry codes: (x) 1/2 - x, 1/2 - y, -z]

**Fig. 3.**
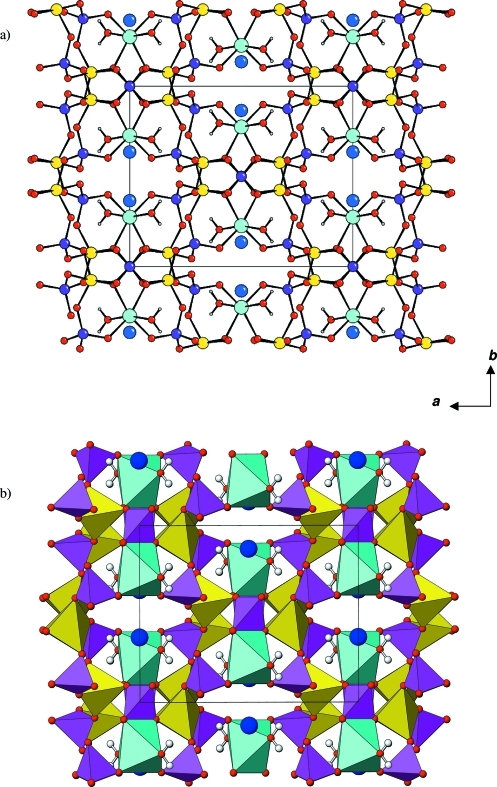
View along the c axis as (a) ball and stick and (b) polyhedral representation showing the elliptical channels bounded by eight membered rings. The shortest cross channel distances are 4.092 (2) and 4.360 (1) Å for O2···O2xi and O2···O6ix respectively. These channels intersect with the main channels shown in Fig.2 to form a 3-D pore network. [Symmetry codes: (ix) -x + 1/2, y + 1/2, z; (xi) -x, 1 - y, z].

## supplementary crystallographic information

### Comment

Although the majority of heterometal substituted gallophosphates, MeGaPOs, contain MO_4_ (*M* = Me or Ga) and PO_4_ units (Baerlocher *et al.*, 2001), some are known in which the metal atoms have non-tetrahedral geometries. For example, NGP-1 (Lin & Wang, 2005), the only organically templated NiGaPO reported to date, contains Ni and Ga disordered over the same octahedral metal sites, together with GaO_5_ and PO_4_ units. K[NiGa_2_(PO_4_)_3_(H_2_O)_2_] is assembled from the same polyhedra as NGP-1 and is isostructural with a number of ammonium transition-metal gallophosphates, NH_4_[MeGa_2_(PO_4_)_3_(H_2_O)_2_] (Me = Mn (Chippindale *et al.*, 1998), Fe, Ni (Bieniok *et al.*, 2008), Co (Chippindale *et al.*, 1996)) and has an aluminium analogue, K[NiAl_2_(PO_4_)_3_(H_2_O)_2_] (Meyer & Haushalter, 1994). The same structure type occurs in Cs[Fe_3_(PO_4_)_3_(H_2_O)_2_] (Lii & Huang, 1995) and NH_4_[CoAl_2_(PO_4_)_3_(H_2_O)_2_] (Panz *et al.*, 1998) and is related to that of (NH_4_)_3_Ga_2_(PO_4_)_3_ (Lesage *et al.*, 2004).

The two phosphorus atoms have tetrahedral geometry (P1, point symmetry C_2v_; P2,1). Unlike in NGP-1, the metal atoms are located in two distinct sites; Ga1 in a GaO_5_ trigonal bipyramid (1) and Ni1 in an NiO_4_(H_2_O)_2_ distorted octahedron (C_2v_), in which the terminal water molecules, H_2_O4, lie *trans* to each other (Fig. 1). Two of the other oxygen atoms in the NiO_6_ unit, O2 and O2^i^, are two coordinate whilst O3 and O3^i^ are three coordinate. As expected, the M—O3 (*M* = Ni, Ga) and P1—O3 bond lengths are the longest M—O and P–O bond lengths observed in the structure. Bond-valence sums (Brese & OKeeffe, 1991) for P1, P2, Ga1 and Ni1 are 4.80, 4.81, 3.22 and 1.93. respectively, which together with the yellow colour of the crystals, confirm the presence of Ni^2+^ in the structure.

The GaO_5_, NiO_6_ and PO_4 _units link together through edge- and vertex-sharing arrangements to give a three-dimensional framework of composition [NiGa_2_(PO_4_)_3_(H_2_O)_2_]^-^. A set of approximately circular channels, running parallel to the [101] direction, contain the water molecules and potassium ions (Fig. 2). A second set of channels, elliptical in shape, run parallel to the *c* axis (Fig. 3) and intersect with the first set to generate a three-dimensional pore network.

The potassium atom lies in a very distorted coordination environment with six near oxygen atoms (K1···O in range 2.865 (1) to 3.072 (1) Å) with two additional O atoms at 3.397 (1) and two at 3.456 (1) Å). H_2_O4 is also involved in hydrogen bonding to Ga—O—P bridging oxygen atoms.

### Experimental

The title compound was prepared hydrothermally from a gel of composition Ga_2_O_3_: NiCl_2_.6H_2_O:10H_3_PO_4_:156H_2_O:0.1Si(OEt)_4_:5KH_2_PO_4 _which was heated at 433 K for 7 d in a Teflon-lined stainless steel autoclave. The solid product was collected by filtration, washed with deionized water and dried in air. The product consisted of large yellow faceted blocks of the title compound, which could be easily separated from unidentified white powder.

### Refinement

Prior to refinement, reflections with I<3σ(I) were omitted. The H atoms of the water molecule, H_2_O4, were located in a difference Fourier map. Their fractional coordinates were refined subject to bond length and angle restraints [O4–H = 0.85 (1) Å, H–O4–H = 109 (5) °] with isotropic displacement parameters fixed at 0.05 Å^2^.

### Crystal data

**Table:**

K[NiGa_2_(PO_4_)_3_(H_2_O)_2_]	*F*(000) = 1080
*M**_r_* = 558.17	*D*_x_ = 3.286 Mg m^−^^3^
Monoclinic, *C*2/*c*	Mo *K*α radiation, λ = 0.71073 Å
Hall symbol: -C 2yc	Cell parameters from 3597 reflections
*a* = 13.2095 (13) Å	θ = 3–32.4°
*b* = 10.1733 (9) Å	µ = 7.27 mm^−^^1^
*c* = 8.8130 (9) Å	*T* = 150 K
β = 107.68 (1)°	Block, yellow
*V* = 1128.4 (2) Å^3^	0.09 × 0.08 × 0.06 mm
*Z* = 4	

### Data collection

**Table:**

Oxford Diffraction Xcalibur diffractometer	1734 reflections with *I* > 2σ(*I*)
graphite	*R*_int_ = 0.018
ω/2θ scans	θ_max_ = 32.6°, θ_min_ = 2.6°
Absorption correction: multi-scan (*CrysAlis RED*; Oxford Diffraction, 2006)	*h* = −15→19
*T*_min_ = 0.54, *T*_max_ = 0.65	*k* = −15→15
3597 measured reflections	*l* = −13→11
1913 independent reflections	

### Refinement

**Table:**

Refinement on *F*	Primary atom site location: structure-invariant direct methods
Least-squares matrix: full	Hydrogen site location: inferred from neighbouring sites
*R*[*F*^2^ > 2σ(*F*^2^)] = 0.022	Only H-atom coordinates refined
*wR*(*F*^2^) = 0.025	Method, part 1, Chebychev polynomial, (Watkin, 1994, *P*rince, 1982) [*w*eight] = 1.0/[A_0_*T_0_(x) + A_1_*T_1_(x) ··· + A_n-1_]*T_n-1_(x)] where A_i_ are the Chebychev coefficients listed belo*w* and x = *F* /*F*max Method = Robust Weighting (*P*rince, 1982) W = [*w*eight] * [1-(delta*F*/6*sigma*F*)^2^]^2^ A_i_ are: 18.9 -12.3 16.6
*S* = 1.04	(Δ/σ)_max_ = 0.001
1667 reflections	Δρ_max_ = 0.84 e Å^−^^3^
103 parameters	Δρ_min_ = −0.91 e Å^−^^3^
3 restraints	

### Fractional atomic coordinates and isotropic or equivalent isotropic displacement parameters (Å^2^)

**Table:**

	*x*	*y*	*z*	*U*_iso_*/*U*_eq_	
Ga1	0.172478 (11)	0.075592 (15)	0.069169 (16)	0.0036	
Ni1	0.0000	0.27385 (3)	0.2500	0.0053	
K1	0.0000	0.63513 (6)	0.2500	0.0264	
P1	0.0000	0.00118 (5)	0.2500	0.0036	
P2	0.20870 (3)	0.37358 (4)	0.17941 (4)	0.0043	
O1	0.05847 (8)	0.09109 (11)	−0.11504 (12)	0.0063	
O2	0.09754 (9)	0.39793 (11)	0.18796 (14)	0.0082	
O3	0.07314 (9)	0.09888 (11)	0.19895 (13)	0.0064	
O4	0.09834 (9)	0.29514 (12)	0.48612 (14)	0.0111	
O5	0.20652 (8)	−0.08860 (10)	0.16197 (12)	0.0061	
O6	0.27707 (8)	0.04382 (11)	−0.04158 (13)	0.0070	
O7	0.23679 (8)	0.22862 (11)	0.15963 (13)	0.0070	
H1	0.135 (3)	0.236 (3)	0.547 (4)	0.0500*	
H2	0.136 (3)	0.361 (3)	0.481 (5)	0.0500*	

### Atomic displacement parameters (Å^2^)

**Table:**

	*U*^11^	*U*^22^	*U*^33^	*U*^12^	*U*^13^	*U*^23^
Ga1	0.00279 (8)	0.00233 (9)	0.00525 (8)	−0.00019 (4)	0.00068 (5)	0.00009 (4)
Ni1	0.00413 (11)	0.00400 (12)	0.00818 (12)	0.0000	0.00228 (8)	0.0000
K1	0.0287 (3)	0.0109 (2)	0.0465 (4)	0.0000	0.0215 (3)	0.0000
P1	0.00246 (18)	0.0030 (2)	0.00514 (19)	0.0000	0.00089 (14)	0.0000
P2	0.00345 (15)	0.00312 (15)	0.00631 (14)	−0.00077 (10)	0.00159 (11)	−0.00031 (10)
O1	0.0048 (4)	0.0058 (4)	0.0066 (4)	0.0003 (3)	−0.0007 (3)	−0.0014 (3)
O2	0.0042 (4)	0.0074 (4)	0.0140 (4)	0.0001 (3)	0.0042 (3)	0.0016 (4)
O3	0.0058 (4)	0.0043 (4)	0.0108 (4)	−0.0002 (3)	0.0051 (3)	0.0010 (3)
O4	0.0106 (5)	0.0083 (4)	0.0115 (5)	−0.0003 (4)	−0.0009 (4)	0.0023 (4)
O5	0.0063 (4)	0.0039 (4)	0.0072 (4)	0.0008 (3)	0.0006 (3)	0.0018 (3)
O6	0.0071 (4)	0.0062 (4)	0.0090 (4)	0.0011 (3)	0.0042 (3)	0.0013 (3)
O7	0.0068 (4)	0.0038 (4)	0.0104 (4)	−0.0017 (3)	0.0029 (3)	−0.0030 (3)

### Geometric parameters (Å, °)

**Table:**

Ga1—O1	1.8556 (11)	P1—O1^ii^	1.5279 (11)
Ga1—O3	1.9994 (11)	P1—O1^iii^	1.5279 (11)
Ga1—O5	1.8541 (10)	P1—O3	1.5452 (11)
Ga1—O6	1.9455 (11)	P1—O3^i^	1.5452 (11)
Ga1—O7	1.8357 (11)	P2—O2	1.5135 (11)
Ni1—O2	1.9951 (11)	P2—O5^iv^	1.5510 (11)
Ni1—O2^i^	1.9951 (11)	P2—O6^v^	1.5348 (12)
Ni1—O3	2.1374 (11)	P2—O7	1.5434 (12)
Ni1—O3^i^	2.1374 (11)	O4—H1	0.850 (10)
Ni1—O4	2.1030 (12)	O4—H2	0.848 (10)
Ni1—O4^i^	2.1030 (12)		
			
O1—Ga1—O3	89.49 (5)	O4^i^—Ni1—O4	168.18 (7)
O1—Ga1—O5	119.09 (5)	O1^ii^—P1—O1^iii^	104.18 (9)
O1—Ga1—O6	94.97 (5)	O1^ii^—P1—O3	114.14 (6)
O1—Ga1—O7	116.97 (5)	O1^iii^—P1—O3	112.42 (6)
O3—Ga1—O5	88.16 (5)	O3^i^—P1—O1^ii^	112.42 (6)
O3—Ga1—O6	174.93 (4)	O3^i^—P1—O1^iii^	114.14 (6)
O3—Ga1—O7	87.06 (5)	O3^i^—P1—O3	99.94 (8)
O5—Ga1—O6	87.57 (5)	O2—P2—O7	115.61 (6)
O5—Ga1—O7	123.66 (5)	O5^iv^—P2—O2	111.12 (6)
O6—Ga1—O7	93.05 (5)	O5^iv^—P2—O6^v^	110.43 (6)
O2^i^—Ni1—O2	101.49 (7)	O5^iv^—P2—O7	101.91 (6)
O2—Ni1—O3	95.65 (4)	O6^v^—P2—O2	107.68 (6)
O2^i^—Ni1—O3	162.84 (5)	O6^v^—P2—O7	110.02 (6)
O2—Ni1—O4	87.12 (5)	Ga1—O1—P1^iii^	135.47 (7)
O2^i^—Ni1—O4	85.41 (5)	Ga1—O3—Ni1	129.51 (5)
O3^i^—Ni1—O2	162.84 (5)	Ga1—O3—P1	131.86 (7)
O3^i^—Ni1—O2^i^	95.65 (4)	Ni1—O2—P2	128.84 (7)
O3^i^—Ni1—O3	67.23 (6)	Ni1—O3—P1	96.42 (5)
O3^i^—Ni1—O4	93.46 (4)	Ni1—O4—H1	128 (3)
O3^i^—Ni1—O4^i^	96.38 (5)	Ni1—O4—H2	103 (3)
O4^i^—Ni1—O2	85.41 (5)	H1—O4—H2	111 (3)
O4^i^—Ni1—O2^i^	87.12 (5)	Ga1—O5—P2^vi^	129.28 (7)
O3—Ni1—O4	96.38 (5)	Ga1—O6—P2^v^	125.62 (7)
O4^i^—Ni1—O3	93.46 (4)	Ga1—O7—P2	139.82 (7)

Symmetry codes: (i) −*x*, *y*, −*z*+1/2; (ii) *x*, −*y*, *z*+1/2; (iii) −*x*, −*y*, −*z*; (iv) −*x*+1/2, *y*+1/2, −*z*+1/2; (v) −*x*+1/2, −*y*+1/2, −*z*; (vi) −*x*+1/2, *y*−1/2, −*z*+1/2.

### Hydrogen-bond geometry (Å, °)

**Table:**

*D*—H···*A*	*D*—H	H···*A*	*D*···*A*	*D*—H···*A*
O4—H1···O5^ii^	0.85 (1)	1.90 (1)	2.741 (2)	172
O4—H2···O6^iv^	0.85 (1)	2.16 (2)	2.976 (2)	161

Symmetry codes: (ii) *x*, −*y*, *z*+1/2; (iv) −*x*+1/2, *y*+1/2, −*z*+1/2.
